# Recognizing the Symptoms of Mental Illness following Concussions in the Sports Community: A Need for Improvement

**DOI:** 10.1371/journal.pone.0141699

**Published:** 2015-11-04

**Authors:** Jane Topolovec-Vranic, Stanley Zhang, Hatty Wong, Emily Lam, Rowan Jing, Kelly Russell, Michael D. Cusimano, Canadian Brain Injury and Violence Research Team

**Affiliations:** 1 Li Ka Shing Knowledge Institute and Trauma and Neurosurgery Program, St. Michael’s Hospital, Toronto, Canada; 2 Faculty of Medicine (Occupational Science and Occupational Therapy), University of Toronto, Toronto, Canada; 3 Division of Neurosurgery, St. Michael’s Hospital, Toronto, Canada; 4 Department of Pediatrics, University of Manitoba, Winnipeg, Canada; 5 Faculty of Medicine and the Dalla Lana School of Public Health, University of Toronto, Toronto, Canada; Zhejiang Key Laborotory for Research in Assesment of Cognitive Impairments, CHINA

## Abstract

**Objective:**

To evaluate the awareness of concussion-related symptoms amongst members of the sports community in Canada.

**Methods:**

A cross-sectional national electronic survey was conducted. Youth athletes, parents, coaches and medical professionals across Canada were recruited through mailing lists from sports-related opt-in marketing databases. Participants were asked to identify, from a list of options, the symptoms of a concussion. The proportion of identified symptoms (categorized as physical, cognitive, mental health-related and overall) as well as participant factors associated with symptom recognition were analyzed.

**Results:**

The survey elicited 6,937 responses. Most of the respondents (92.1%) completed the English language survey, were male (57.7%), 35–54 years of age (61.7%), with post-secondary education (58.2%), or high reported yearly household income (>$80,000; 53.0%). There were respondents from all provinces and territories with the majority of respondents from Ontario (35.2%) or British Columbia (19.1%). While participants identified most of the physical (mean = 84.2% of symptoms) and cognitive (mean = 91.2% of symptoms), they on average only identified 53.5% of the mental health-related symptoms of concussions. Respondents who were older, with higher education and household income, or resided in the Northwest Territories or Alberta identified significantly more of the mental health-related symptoms listed.

**Interpretation:**

While Canadian youth athletes, parents, coaches and medical professionals are able to identify most of the physical and cognitive symptoms associated with concussion, identification of mental health-related symptoms of concussion is still lagging.

## Introduction

Concussions occur at least 1.75 million times a year in North America and account for approximately 75% of all traumatic brain injuries (TBI) [1–3]. Sport-related concussion is the most common type of TBI in youth and sports account for over half of the concussions sustained by youth each year. In North America more than a half million youth under the age of 15 who sustain a concussion require hospital-based care each year [4]. This is particularly problematic for young people because of the potential cumulative or long term deleterious effects of concussion [5–10]. The Centre for Disease Control recently declared that sport concussions are reaching “epidemic levels” and deserve further research [1].

Concussion can adversely affect a person’s cognitive, emotional and social functioning, and create lasting personal, familial and societal implications. In approximately 10–15% of individuals who have sustained concussion or mild TBI, persistent symptoms can impact an individual’s ability to return to daily functioning [11]. Physical symptoms (headache, nausea, fatigue and dizziness), cognitive symptoms (memory, attention and executive function impairments), and mental health concerns (depression, anxiety and post-traumatic stress disorder) are commonly observed and can negatively impact upon an individual’s ability to recover from concussion [12]. Studies have indicated that 22–36% of patients report three or more of such posttraumatic symptoms at 6 months post mild TBI [13] with about 23% not being recovered by 1 year post-injury [14].

Despite the high incidence and adverse outcomes associated with concussion, studies suggest that knowledge regarding the symptoms associated with concussion is generally low amongst athletes [15–18], coaches and trainers [19, 20] and the general public [21, 22]. Adequate knowledge regarding the identification, treatment and management of concussion-related symptoms is important in order to optimize recovery and decrease the risk of long-term brain injuries [23]. However, studies suggest that there are also significant gaps between physician practices and consensus-based concussion management guidelines [24]. These knowledge gaps may be partly explained by the general lack of concussion-related training in medical school curricula [25].

Recognizing the burden of concussion in Canada, the Public Health Agency of Canada (PHAC) commissioned a national electronic survey as part of a program to raise awareness of concussions to assess concussion-related awareness and knowledge across Canada in order to inform the development and implementation of programs and interventions. The survey was designed to evaluate awareness and knowledge of concussion amongst youth athletes, parents, coaches and medical professionals in Canada. We report the findings related to symptom identification and respondent factors associated with higher proportions of symptom identification.

## Methods

### Ethics and Participants

Contacts of the Coaching Association of Canada (CAC) opted in to receive e-mail communication and the survey link was sent to them along with the rules and regulations of participation. Returning the questionnaire implied consent to participate. Field Day Inc. collated the data and provided the anonymized unidentified dataset to the research team. Therefore, the responses provided by individuals who completed the survey could not be identified and the analysis was performed on the aggregated data. This consent procedure and the study were approved by St. Michael’s hospital’s Research Ethics Board. Data were derived from a national electronic survey conducted in April, 2012. The survey was comprised of ten true/false and multiple choice items which were designed to assess the awareness and understanding of concussion amongst the general Canadian public and within the sport community. The survey items included: knowledge of concussion symptoms, experience with concussion, attitude towards concussion and demographic information.

### Primary Outcome: Concussion Symptom Recognition

The present questionnaire used in the survey was modified from a previously validated questionnaire [15]. It was developed with both medical and sports experts and went through several pilot forms to maximize accuracy, comprehensiveness and readability before use. It also had a high internal consistency as measured by cronbach's alpha (0.87 for the overall questionnaire and 0.86–0.88 for each individual question). As with any questionnaire more evidence for validation‎ will occur over time.

The survey included a list of 17 symptoms and participants were asked to indicate “What are the signs and symptoms of a concussion (check all that apply)?”. A yes or no response option was provided for each symptom. For analyses, the symptoms were grouped into the following categories: physical, 11 items (loss of consciousness, blurred vision, seizures or convulsions, nausea or vomiting, dizziness, headaches, drowsiness or fatigue, neck pain, balance problems, light sensitivity, sound sensitivity); cognitive, 3 items (confusion, problems remembering, problems concentrating); and mental health-related, 3 items (nervousness or anxiety, sadness or depression, irritability).

### Setting: Survey Administration

The survey was conducted online by an independent Canadian agency, Field Day Inc., and was hosted by surveymonkey.com. Awareness of the survey was promoted via a variety of methods such as: social media, contest incentives, Field Day’s website and three mass e-mailings. One mass e-mail was sent to a database of Canadian adults who opted in to receive emails related to surveys and contests (managed by Field Day); a second mass e-mail went to the Coaching Association of Canada's opt-in marketing database and a third mass e-mail was sent through the True Sport Movement opt-in marketing database. In addition, the survey link was distributed through partner listservs/networks. As an incentive, participants were offered a chance to win one tablet computer donated by Field Day Inc.

Two versions of the survey were created: one in English and one in French. The opening page of the survey provided a bilingual overview of the survey (purpose, approximate length of time required to complete the survey, contest details). The survey was reviewed and approved by the St. Michael’s Hospital Research Ethics Board.

### Data Analyses

Statistical analyses were performed using SAS 9.3 (Chicago, USA). Respondents were instructed to check off yes or no for each of the 17 listed symptoms of concussion. To determine the degree of concussion symptom identification, the percentage of all respondents indicating yes for each symptom was calculated. Also, the proportions of all symptoms identified across all respondents in each of the categories (physical, cognitive, mental health or overall) were calculated. Although the proportions for physical and cognitive symptoms were not normally distributed, the results are shown as means and 95% confidence intervals based on the assumptions of the central limit theorem for large sample sizes [26].

To determine if there were differences in symptom identification proportions across the three groups of symptoms (physical, cognitive, mental health) an ANOVA (for normally distributed data) or non-parametric tests (Kruskal –Wallis H test or Wilcoxon rank sum test for non-normally distributed data) were conducted. These same tests were used to assess whether there were differences in symptoms identification proportions across demographic variables (survey language, gender, age, education level, geographic location and self-identified primary role). Post-hoc tests using the least significant difference t-test or Nemenyi test were conducted to identify which comparisons were significantly different.

## Results

### Survey Respondents

Overall, 7258 people started and 6,937 people completed the survey (95.6% completion rate). As detailed in Table 1 most of the respondents (92.1%) completed the English language survey, were male (57.7%), 35–54 years of age (61.7%), with post-secondary education (58.2%), or high reported yearly household income (>$80,000; 53.0%). The majority of respondents identified themselves as a coach or trainer (73.5%). There were respondents from all provinces and territories with the highest proportions from Ontario (35.2%) or British Columbia (19.1%).

**Table 1:** Demographic characteristics of the survey respondents (n = 6937).

Characteristic		Number of respondents (%)
Survey language	English	6391 (92.1)
	French	546 (7.9)
Gender	Male	4001 (57.7)
	Female	2890 (41.7)
	Prefer not to say	46 (0.7)
Age, years	Under 15	4 (0.1)
	15–24	562 (8.1)
	25–34	1041 (15)
	35–44	1880 (27.1)
	45–54	2397 (34.6)
	55–64	811 (11.7)
	65–74	158 (2.3)
	75+	16 (0.2)
	Prefer not to say	68 (1.0)
Yearly household income	$0-$20,000	221 (3.2)
	$20,001-$40,000	414 (6)
	$40,001-$60,000	860 (12.4)
	$60,001-$80,000	73 (1.1)
	$80,001-$100,000	1377 (19.9)
	$100,001-$150,000	1366 (19.7)
	$150,001+	929 (13.4)
	Prefer not to say	1697 (24.5)
Education	Some high school	45 (0.7)
	High school	405 (5.8)
	Some post-secondary	860 (12.4)
	Post-secondary (college/university)	4034 (58.2)
	Master’s Degree	798 (11.5)
	Doctoral Degree	157 (2.3)
	Professional Designation	495 (7.1)
	Other	69 (1)
	Prefer not to say	74 (1.1)
Role^a^	Athlete	3464 (48.4)
	Parent or guardian of athlete	3836 (53.6)
	Coach^b^ or trainer	5258 (73.5)
	Team, club, or organization personnel	1920 (26.8)
	Educator, teacher or learning facilitator	1598 (22.3)
	Medical professional	509 (7.1)
	Other	451 (6.5)
Location	Ontario	2444 (35.2)
	British Columbia	1326 (19.1)
	Alberta	1034 (14.9)
	Quebec	622 (9.0)
	Manitoba	447 (6.5)
	Saskatchewan	294 (4.2)
	New Brunswick	272 (3.9)
	Nova Scotia	255 (3.7)
	Newfoundland& Labrador	91 (1.3)
	Outside Canada	51 (0.7)
	Prince Edward Island	39 (0.6)
	Northwest Territories	33 (0.5)
	Yukon	23 (0.3)
	Nunavut	6 (0.1)

^a^Respondent could indicate up to 3 categories that best described their position.

^b^Includes community coach, competitive coach, and high performance coach.

### Concussion Symptom Recognition

**Fig 1:**
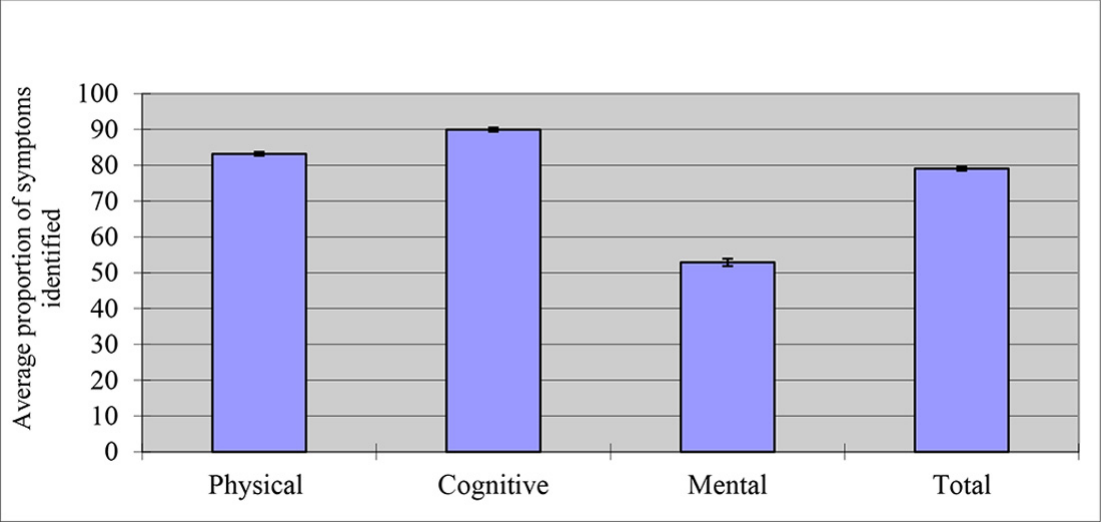
Average proportion of concussion-related symptoms identified by survey respondents. F = 2982.24, p < .0001 Post-hoc tests were significant among pair-wise comparisons of physical, cognitive and mental symptoms.

Fig 1 summarizes the results of respondents’ identification of concussion-related symptoms. On average, the respondents identified 84.2% (95% CI: 83.8%-84.7%) of the 11 listed physical symptoms associated with concussion. Most of the respondents identified headaches (98.5%) or dizziness (96.3%) with the fewest respondents identifying neck pain (63.7%) or seizures/convulsions (69.0%).

Similarly, respondents identified on average 91.2% (95% CI: 90.6%-91.7%) of the 3 listed cognitive symptoms with most participants (95.0%) identifying confusion problems as being associated with a concussion. In contrast, respondents on average only identified 53.5% (95% CI: 52.5%-54.6%) of the 3 listed mental health-related symptoms of concussions. The highest proportion of respondents (59.4%) identified irritability and the fewest identified nervousness or anxiety (50.6%)(Table 2).

**Table 2:** Percentage of survey respondents to identified each of the listed physical, cognitive, and mental-health related symptoms (n = 6937).

	Signs or symptoms	Number of persons identifying the symptom (%)
Physical symptoms	Headaches	6831 (98.5)
	Dizziness	6677 (96.3)
	Blurred vision	6577 (94.8)
	Nausea or vomiting	6437 (92.8)
	Loss of consciousness	6379 (92.0)
	Balance problems	5930 (85.5)
	Drowsiness or fatigue	5918 (85.3)
	Light sensitivity	5497 (79.2)
	Sound sensitivity	4821 (69.5)
	Seizures or convulsions	4787 (69.0)
	Neck pain	4417 (63.7)
Cognitive symptoms	Confusion	6588 (95.0)
	Problems concentrating	6215 (89.6)
	Problems remembering	6169 (88.9)
Mental-health symptoms	Irritability	4119 (59.4)
	Sadness or depression	3512 (50.6)
	Nervousness or anxiety	3508 (50.6)

With respect to the total number of symptoms identified, those who completed the French language survey, were 35–54 years of age, had higher education and household income, or were in the medical professional identified the highest proportion of symptoms (Table 3). Similar patterns were observed for each of the sub-groups of physical, cognitive and mental health symptoms with the following differences: the French language survey respondents identified the same proportion of physical and cognitive symptoms as the English language survey respondent; female respondents identified more mental health related symptoms than the male respondents.

**Table 3:** Average proportion of symptoms (95% CI) identified by demographic characteristic (n = 6937).

Characteristic	Average Proportion of Symptoms Identified			
	Physical	Cognitive	Mental Health	Total
**Survey language**				
English ^C^	84.2(83.7,84.7)	91.1(90.6,91.7)	52.4(51.4,53.5)	79.8(79.3,80.3)
French	84.4(82.5,86.3)	91.3(89.5,93.2)	66.1(62.3,69.9)*	82.4(80.3,84.4)*
**Gender**				
Female^C^	84.4(83.7,85.0)	91.6(91.0,92.3)	55.5(54.2,56.9)	80.6(79.9,81.2)
Male	84.1(83.4,84.8)	90.6(89.7,91.4)	50.9(49.3,52.5)*	79.4(78.6,80.1)
Prefer not to say	80.2(73.0,87.4)*	88.4(81.1,95.7)*	42.8(30.1,55.4)*	75.1(67.7,82.4)*
**Age, years**				
Under 15	72.7(41.9,100)	66.7(28.9,100)*	50.0(0,100)*	67.6(0,100)*
15–24^C^	81.5(79.8,83.3)	88.6(86.5,90.7)	44.8(41.2,48.5)	76.3(74.5,78.1)
25–34	84.4(83.2,85.5)*	89.8(88.4,91.2)	50.7(48.0,53.4)	79.4(78.1,80.6)*
35–44	85.0(84.2,85.9)*	91.6(90.6,92.6)*	55.8(53.8,57.7)*	81.0(80.1,81.9)*
45–54	85.2(84.4,85.9)*	92.2(91.3,93.0)*	56.2(54.5,58.0)*	81.3(80.5,82.1)*
55–64	82.3(80.9,83.8)	90.9(89.4,92.4)	51.0(48.0,54.1)*	78.3(76.8,79.8)
65–74	80.1(77.0,83.3)	91.4(88.0,94.7)	49.8(43.0,56.5)	76.8(73.5,80.0)
75+	76.7(63.4,90.1)	87.5(73.2,100.0)	54.2(30.0,78.3)	74.6(61.9,87.4)
Prefer not to say	83.8(79.5,88.2)*	91.7(86.8,96.6)*	50.0(39.6,60.4)*	79.2(74.5,84.0)*
**Education completed**				
Some high school^C^	67.3(60.5,74.0)	76.3(66.6,86.0)	25.2(14.9,35.5)	61.4(54.8,68.1)
High school	79.9(77.8,82.0)*	87.9(85.5,90.3)*	42.0(37.6,46.3)*	74.6(72.4,76.8)*
Some post-secondary	83.2(81.8,84.5)*	90.2(88.7,91.7)*	49.6(46.6,52.6)*	78.5(77.1,79.9)*
Post-secondary (college/university)	84.3(83.7,84.9)*	91.0(90.3,91.7)*	53.4(52.0,54.7)*	80.0(79.4,80.7)*
Professional Designation	85.8(84.5,87.1)*	93.0(91.6,94.4)*	59.6(56.6,62.6)*	82.4(81.0,83.8)*
Master’s Degree	84.4(81.2,87.5)*	92.8(89.5,96.1)*	60.7(54.0,67.5)*	81.7(78.4,85.0)*
Doctoral Degree	87.6(86.0,89.1)*	94.7(93.1,96.4)*	60.8(57.0,64.6)*	84.1(82.5,85.8)*
Other	85.5(81.2,89.8)*	92.3(87.3,97.4)*	57.2(46.9,67.5)*	81.7(76.9,86.6)*
Prefer not to say	84.2(79.6,88.8)*	90.3(84.7,96.0)*	55.1(44.8,65.3)*	80.1(75.3,85.0)*
**Yearly householdincome**				
$0-$20,000^C^	78.4(75.3,81.6)	86.3(82.7,89.9)	43.0(37.2,48.8)	73.5(70.3,76.8)
$20,001–40,000	80.5(78.3,82.6)	88.0(85.5,90.5)	45.8(41.5,50.1)	75.7(73.5,77.9)
$40,001–60,000	83.3(82,84.7.0)*	90.3(88.8,91.9)*	51.7(48.8,54.7)*	79.0(77.6,80.4)*
$60,001–80,000	82.3(77.8,86.8)	92.2(87.7,96.8)*	47.0(36.6,57.5)	77.8(73.1,82.5)
$80,001–100,000	83.9(82.9,84.9)*	90.6(89.4,91.7)*	52.4(50.0,54.7)*	79.5(78.4,80.6)*
$100,001–150,000	86.4(85.5,87.3)*	92.6(91.5,93.7)*	58.3(56.0,60.6)*	82.5(81.5,83.6)*
$150001+	87.3(86.2,88.4)*	94.4(93.2,95.6)*	60.2(57.5,63.0)*	83.8(82.6,85.0)*
Prefer not to say	83.3(82.3,84.2)*	90.5(89.4,91.6)*	51.4(49.3,53.5)*	78.9(77.9,79.9)*
**Role** ^a^				
Athlete	85.6(84.9,86.2)#	92.4(91.7,93.1)#	54.9(53.5,56.4)#	81.3(80.7,82.0)#
Parent or guardian of athlete	85.9(85.3,86.4)#	92.4(91.8,93.1)#	56.3(54.9,57.7)#	81.8(81.2,82.4)#
Coach^b^ or trainer	85.3(84.8,85.8)#	92.4(91.8,93.0)#	55.1(53.9,56.4)#	81.2(80.6,81.8)#
Team, club, or organizationpersonnel	86.2(85.4,87.1)#	93.5(92.7,94.4)#	56.7(54.8,58.6)#	82.3(81.5,83.2)#
Educator, teacher or learning facilitator	86.9(86.1,87.8)#	93.9(93.0,94.8)#	57.3(55.2,59.4)#	82.9(82.0,83.9)#
Medical professional	89.3(88.0,90.6)#	96.7(95.6,97.9)#	70.3(66.9,73.6)#	87.3(85.8,88.7)#
Other	83.3(81.4,85.3)#	89.9(87.7,92.1)#	52.2(48.0,56.3)#	79.0(76.9,81.1)#
**Location**				
Alberta^C^	86.6(85.6,87.7)	92.3(91.1,93.6)	58.2(55.5,60.8)	82.6(81.4,83.8)
British Columbia	84.7(83.7,85.7)*	92.3(91.1,93.4)	54.9(52.6,57.3)	80.8(79.7,81.8)*
Manitoba	85.2(83.5,87.0)	92.4(90.5,94.3)	51.8(47.6,55.9)*	80.6(78.7,82.5)
New Brunswick	85.8(83.6,87.9)	92.6(90.3,94.9)	51.2(46.0,56.5)*	80.9(78.6,83.2)
Newfoundland & Labrador	84.0(80.2,87.8)	91.2(86.6,95.8)	51.3(41.5,61.1)	79.5(75.1,83.9)
Northwest Territories	88.4(83.3,93.6)	88.9(80.2,97.6)	59.6(43.8,75.4)	83.4(76.9,90.0)
Nova Scotia	84.4(82.3,86.5)	92.8(90.2,95.4)	53.1(47.9,58.2)	80.3(78.1,82.6)
Nunavut	56.1(22.8,89.3)*	55.6(13.2,97.9)*	33.3(0,77.6)	52.0(16.7,87.2)*
Ontario	83.2(82.4,84.0)*	89.6(88.6,90.5)*	50.6(48.8,52.3)*	78.5(77.7,79.4)*
Outside Canada	80.2(74.3,86.1)*	88.9(82.5,95.3)*	47.1(35.3,58.8)	75.9(69.7,82.1)*
Prince Edward Island	84.8(80.0,89.7)	96.6(91.2,100.0)	42.7(28.7,56.7)*	79.5(73.9,85.0)
Quebec	82.9(81.1,84.6)*	90.8(89.1,92.6)	60.1(56.5,63.7)	80.2(78.4,82.1)*
Saskatchewan	83.3(81.1,85.6)*	91.8(89.4,94.3)	50.1(45.0,55.2)*	79.0(76.6,81.3)*
Yukon	81.4(73.8,89.1)*	95.7(89.1,100.0)*	49.3(32.0,66.6)*	78.3(70.2,86.3)*

^a^Respondent could indicate up to 3 categories that best described their position.

^b^Includes community coach, competitive coach, and high performance coach.

^c^Comparison group.

*p<0.05 as compared with the comparison group.

^#^p<0.05 as compared with all other roles in the group combined.

There were some geographical variances in symptom recognition with respondents from Alberta and the Northwest Territories identifying the most total number of symptoms (82.6% and 83.4%, respectively). These same respondents identified the most physical symptoms (Alberta, 82.6%; Northwest Territories 83.4%). Respondents from the Yukon and Prince Edward Island identified the most cognitive symptoms of concussion (95.7% and 96.6%, respectively) whereas those from the Northwest Territories and Quebec identified most of the mental health-related symptoms (59.6% and 60.1%, respectively). Medical professionals identified the highest proportion of all the symptoms (89.3% for physical symptoms, 96.7% for cognitive symptoms and 70.3% for mental health-related symptoms; Table 3). Coaches and trainers from competitive and non-competitive levels recognized a comparable number of symptoms as other respondents (e.g. athlete, parents or guardian of athletes) (Table 3).

## Discussion

Unlike previous studies which have suggested that the concussion knowledge of the coaches, athletic trainers and the general public is generally poor [15, 19, 20] our cross-Canada study has shown that most people engaged in the sports community are familiar with the physical and cognitive symptoms associated with concussion. Also, the studies were published in 2007–2009 and concussion has received notable media attention since then. Despite finding higher levels of overall concussion symptom knowledge, our study highlights a potential gap in knowledge and awareness related to the mental health outcomes of concussion with respondents identifying, on average, only half of such symptoms (nervousness or anxiety, sadness or depression and irritability) as possible symptoms of concussion. This gap is significant as studies suggest a positive association between childhood mild TBI and the risk of subsequent psychiatric diagnoses [27]. In a study of 42 children with mild TBI admitted to the hospital, 35.7% had mood disorders and 21.4% had anxiety disorders 6 months following their injury [28]. A study of nearly 5,000 adolescents across Ontario has shown that adolescents who have sustained TBI where they lost consciousness for at least 5 minutes or were hospitalized due to their TBI were at significantly greater risk of psychological distress, suicide attempts, being prescribed medication for anxiety, depression or both, as well as being victimized through bullying, cyber-bullying, or being threated with a weapon at school [29]. Moreover, a retrospective cohort study of over 36,000 adolescents in the US indicated that a history of concussion was associated with a 3.3-fold greater risk (95% CI: 2.0–5.5) for subsequent depression diagnosis prompting the authors to conclude that clinicians should screen for depression amongst youth who have sustained concussion to enable effective treatment [27]. The physical, cognitive and mental health-related symptoms of concussion occur at different rates and last for various periods of time and thus constitute an important diagnostic criterion of postconcusion syndrome for both ICD10 and DSM-IV. However, such symptom occurrence rates vary drastically depending on studies. For instance, Oddy et al (1978) reported that 20.8%, 37.5%, and 33% of the studied cohort had depression, memory problem, and fatigue, respectively[30], while only 8% had memory problems and 11% had fatigue in Ettenhofer’s study [31]. Gerberich et al (1983) found that 14% of the 3063 secondary school varsity football players had blurred/double vision [32]. In contrast to these rates, recent study by Grubenhoff et al (2011) showed that the rate for depression, fatigue, and blurred/double vision in concussed children with altered mental state is 24%, 82%, and 39%, respectively [33]. The rate variations are likely from a number of sources, such as the studied cohorts, the time of study subjects taking questionnaires related to the time course of their concussion, the mechanisms of the injury, the type of sports, etc.

Although the occurrence rate of physical, cognitive and mental health-related symptoms of concussion fluctuated, one can still argue that it might directly affect neither one’s concussion knowledge nor his ability to make the accurate diagnosis of concussion provided that the individual had adequate knowledge of concussion. Although the occurrence of concussion symptoms changes, survey respondents’ knowledge is reflected and thus can be measured by their ability to correctly identify the right symptoms. This same approach of measurement has also been used to assess the public knowledge of the symptoms of myocardial infarction [34]. The authors don’t believe that the number of survey symptoms itself has any effects on the results, although the number of symptoms in some categories is small. Almost the same small number of questions can be found in subscales of SF-36, a well validated and widely used questionnaire [35].

These findings stress the need for educational initiatives to increase awareness regarding mental-health related symptoms associated with concussion. Prompt recognition of mental health disorders following TBI is necessary given their frequency and potential to impede recovery in other symptom areas [36]. For example, among adults with no psychiatric diagnoses in the year preceding mild TBI, there was an almost 3-fold risk of a psychiatric diagnosis in the first 6 months post-mild TBI as compared with a non-injured adult control group which persisted through the first two years post-injury [37]. A lower level of concussion knowledge has been shown to be related to higher post-concussion symptom severity [27] and the early provision of education has been shown to reduce symptom severity [29], [36]. The extent to which the effectiveness of early education is related to factual knowledge about concussion rather than positive expectancies is uncertain, and the extent to which mental health related information is included in the educational initiatives is unknown.

The recognition of symptoms appeared to vary geographically with identification of the proportion of symptoms listed ranging from 52.0% (Nunavut) to 83.4% (Northwest Territories). The exact reasons are unknown. It is tempting to speculate that provinces and regions with more accessible concussion educational resources or campaigns may provide their residents some advantages in knowing and even managing concussion. The exact reasons are a topic of future research to explore and identify. Our findings provide direction as to potential geographic areas where concussion awareness strategies could be focussed, for example through engagement with the local media. Blake and colleagues reported that evidence-based messages regarding the clinical and public health significance of concussion and the impact of body checking in youth ice hockey were accurately reported in English-language Canadian newspaper coverage [38] supporting the potential use of media as a vehicle for knowledge translation to aide in injury prevention strategies [27]. Whether such coverage improves concussion-related knowledge amongst readers needs to be evaluated.

Self-identified medical professionals identified the highest proportion of the listed concussion-related symptoms. Surprisingly, our survey showed that individuals who self-identified as coaches and trainers were no better able to recognize concussion related symptoms than athletes (total symptoms: coach/trainer 81.2%; sports related personnel 82.3%; athlete 81.2%), particularly mental-health related symptoms (coach/trainers 55.1%; sports related personnel 56.7%; athletes 54.9%). This is worrisome as coaches are often the first line responders in cases of suspected concussion, and it is their decisions on the field (to return the athlete to the game or not) which can have dramatic and long-lasting repercussions upon the health and recovery of the athlete. Educational initiatives such as workshops about concussion geared at coaches have been shown to effectively improve their concussion-related knowledge [[17, 18]]. While Cusimano et al. reported significantly increased concussion knowledge scores amongst athletes following viewing of an educational video, the increased knowledge was not sustained at a two-month follow-up [15–18] highlighting the need for strategies to enhance knowledge retention. Studies also support the notion of multifaceted approaches such as education combined with rule changes, changes in the built environment and better use of protective equipment to prevent concussion [39, 40].

Some concussion resolve quickly and others leave devastating consequences such as disability, chronic traumatic encephalopathy, or even death. To date, the diagnosis of concussion is almost completely relying on its symptoms. Hence, an individual’s ability to recognize such symptoms determines how quickly they seek medical attention while knowing the clinical consequences of concussion allows them to decide whether or not they take concussion seriously. Therefore, educating the general public and raising their awareness of concussion is key not only to the prevention of concussion, but also to its timely management where time is critically important to the clinical outcomes. In addition, psychiatric consequences of concussions are becoming more and more recognized and investigated. Searching Pubmed for “psychiatric symptoms of concussion” yielded only 9 publication from 1940–1969 compared to 10 articles from 1970–1979, 35 from 1980–1989, 61 from 1990–1999, and 85 from 2000–2009. The same search resulted in 138 publications in the last five years alone, indicating that a) researchers start to study this symptom domain that is often overlooked; b) more people realize the importance of the mental health aspects of concussion. These study results, including the findings of the present study, would be relevant to clinicians (e.g. better diagnose and treat concussion), athletes and athletic communities (e.g. seek medical services in a timely manner), teachers/employers (e.g. follow return-to-school or return-to-work guidelines), family members (e.g. support concussed person to deal with physical, cognitive, emotional, or psychiatric symptoms and dysfunctions). To this end, knowing the awareness of the public is indeed of paramount significance in public health, medicine, sport sciences, and medical education.

### Limitations

Strengths of this study include the large national sample and the concurrent assessment of various groups including athletes, parents, coaches and medical professionals. Although our sample size was large and national in scope, our findings are limited by evaluating group level data rather than individual-level data. Also, the results were derived from self-reported knowledge, rather than a test of concussion-related knowledge (e.g. through case studies or multiple choice responses). Finally, future research is needed to determine whether the ability to recognize symptoms of concussion translates into the prevention of future injuries, or better management of sustained injuries.

## Conclusions

In our large national survey of athletes, parents, coaches and medical professionals a high number of the physical and cognitive symptoms associated with concussion were identified. However, significantly fewer respondents associated the mental health symptoms of nervousness or anxiety, sadness or depression, and irritability with concussion. These findings help to shed light on areas in which concussion awareness and knowledge initiatives need to be targeted to enhance the prevention and recovery from sport-related concussion.

## Supporting Information

S1 Supporting Information Members of the Canadian Brain Injury and Violence Research Team. (DOCX)

S2 Supporting Information Concussion survey for 2012. (DOCX)
