# Supplementary material for: Recognizing the Symptoms of Mental Illness following Concussions in the Sports Community: A Need for Improvement
Source: PLoS One. 2015 Nov 4;10(11):e0141699. doi: 10.1371/journal.pone.0141699 (PMC4633152; doi:10.1371/journal.pone.0141699)
Supplement: S1 Supporting Information — (DOCX) [file pone.0141699.s001.docx]

# Members of the Canadian Brain Injury and Violence Research Team

Members of the Canadian Brain Injury and Violence Research Team include Blaine Hoshizaki, Robert Mann, Tom Schweizer, David Wolfe, Mark Asbridge, Shree Bhalerao, David Clarke, Angela Colantonio, Paul Comper, Wendy Cukier, Eric Vaz, Jim Cullen, David Delay, Peter Donnelly, Simon Graham, Jeff Hoch, Stephen Hwang, Claus Rinner, Eric Roy, Aron Shlonsky, Charles Tator, Lorne Tepperman, Jane Topolovec-Vranic, Donald Voaklander, Michael Huchison, Gabriela Ilie, Rowan Jing, Caroline Lewis, Martyna Krezel, Christopher Pauley, Windsor Ting.
