# Supplementary material for: Recognizing the Symptoms of Mental Illness following Concussions in the Sports Community: A Need for Improvement
Source: PLoS One. 2015 Nov 4;10(11):e0141699. doi: 10.1371/journal.pone.0141699 (PMC4633152; doi:10.1371/journal.pone.0141699)
Supplement: S2 Supporting Information — (DOCX) [file pone.0141699.s002.docx]

**INTRO**

Thank you for your participation in this survey. Your opinions are important, and will help to shape a national program to raise awareness and knowledge about sports-related concussions.

The survey should take no more than 10 minutes of your time.

At the end of the survey, you will have the opportunity enter a contest to win a 16GB iPad 2 with Wi-Fi + 3G with a retail value of $649. This contest is open to residents of Canada, excluding the Province of Quebec, who are over the age of majority in the province or territory in which they reside at the time of the contest entry.

1. Yes, I’d like to take the survey in English
2. Yes, I’d like to take the survey in French

**SECTION 1 – GENERAL KNOWLEDGE**

1. What is a concussion (check all that apply)?
   1. A bruise to the brain
   2. A black out or loss of consciousness
   3. A traumatic brain injury
   4. Whiplash
2. What can cause a concussion (check all that apply)?
   1. A direct bump or blow to the head
   2. Activity that causes a sudden increase in heart rate
   3. A hit to the body that causes the head to move rapidly
   4. Any other activity that causes the head to move rapidly
3. How do you identify a concussion (check all that apply)?
   1. By looking at a CT or MRI scan of the brain
   2. By watching for specific symptoms
   3. By attempting to walk in a straight line with your arms extended
   4. By checking your heart rate and blood pressure
4. What are the signs and symptoms of a concussion (check all that apply)?
   1. Loss of consciousness
   2. Blurred vision
   3. Seizures or convulsions
   4. Nausea or vomiting
   5. Dizziness
   6. Headaches
   7. Confusion
   8. Drowsiness or fatigue
   9. Nervousness or anxiety
   10. Sadness or depression
   11. Irritability
   12. Neck pain
   13. Balance problems
   14. Problems remembering
   15. Problems concentrating
   16. Light sensitivity
   17. Sound sensitivity
5. How do you reduce the likelihood of getting a concussion (check all that apply)?
6. Wear a properly fitted helmet
7. Avoid high-contact situations
8. Avoid high-contact sports until the age of 18
9. Increased level of physical fitness, including cardiovascular and strength training
10. What should you do immediately after noticing symptoms of a concussion (check all that apply)?
    1. Rest for a few minutes until the effects wear off, then resume your activity
    2. Tell a friend, family member, teammate, trainer or coach that you think you might have a concussion
    3. Remove yourself from the sport or activity
    4. Seek medical attention immediately
11. What is the appropriate treatment for a concussion (check all that apply)?
    1. Mental and physical rest until symptom free
    2. Prescribed medication
    3. Psychiatric evaluation
    4. Physiotherapy
12. After a concussion, when should you return to sports (check all that apply)?
    1. As soon as the immediate effects wear off
    2. After you have completed a 6 step protocol with gradual and monitored activity
    3. After you have been cleared by your doctor
    4. Never

**SECTION 2 – FIRST-HAND EXPERIENCE**

1. Have you, a family member or a friend had a sport-related brain injury or concussion?
   1. Yes (If yes, continue to Section 2, Question 2)
   2. No (If no, skip to Section 3)
2. How many diagnosed brain injuries/concussions have you/they had?
3. (enter a number)
4. When did the most recent injury occur?
   1. Within the last month
   2. Within the last year
   3. More than a year ago
5. How did the most recent injury occur?
   1. Direct contact to the head by another player or participant
   2. Impact to the head on the playing surface or boards due to physical contact from another player or participant
   3. Impact to the head not due to physical contact from another player (e.g. a slip or fall)
   4. A jolt to the body without direct impact to the head
   5. Other (please specify)
6. How did you/they identify that it was a brain injury/concussion (check all that apply)?
   1. Experienced signs or symptoms that were out of the ordinary
   2. Evaluation by a coach, trainer or other sport organization personnel
   3. Evaluation by your GP/family doctor
   4. Evaluation by a neurologist or other specialist
   5. Other (please specify)
7. How long after the injury did you/they see a doctor?
   1. The same day
   2. Within one week
   3. Within one month
   4. After one month
   5. Not at all
8. How did you/they follow up and/or treat the injury (check all that apply)?
   1. Physical and mental rest
   2. Avoidance of noise and bright light
   3. Following the 6 step “return to play” protocol
   4. Checkups with family doctor
   5. Consultation with neurologist or other specialist
   6. Other (please specify)
9. How long did the symptoms last?
   1. Less than a week
   2. Less than a month
   3. 1-6 months
   4. 6-12 months
   5. Longer than a year
   6. Still experiencing symptoms
10. How long after the injury did you/they return to participation in the activity?
    1. Less than a week
    2. Less than a month
    3. 1-6 months
    4. 6-12 months
    5. Longer than a year
    6. Have not returned to the activity
11. What might have assisted you in better dealing with the injury (check all that apply)?
    1. Online access to symptom and treatment information
    2. Online access to medical resources (e.g. contact information for neurologists/specialists)
    3. Mandatory training of athletes by the team coach, trainer or other official
    4. Better knowledge about brain injuries/concussion symptoms and treatment by coach, trainer, or other sport organization personnel
    5. Better knowledge about brain injuries/concussions symptoms and treatment by GP/family doctor
    6. Other (please specify)

**SECTION 3 – ATTITUDES TOWARDS CONCUSSION**

1. In your opinion, in which sports/activities are participants most likely to get a brain injury/concussion (rank each sport/activity on a scale from highly unlikely to highly likely)?
   1. Alpine skiing
   2. Baseball
   3. Basketball
   4. Curling
   5. Field hockey
   6. Football
   7. Ice hockey
   8. Lacrosse
   9. Ringette
   10. Rugby
   11. Soccer
   12. Other
2. Does the risk of concussion influence your decision when enrolling in a sport or activity?
   1. Not applicable
   2. No influence
   3. Slight influence
   4. Moderate influence
   5. High influence
3. Does the risk of concussion influence your decision to enroll your child in a sport or activity?
   1. Not applicable
   2. No influence
   3. Slight influence
   4. Moderate influence
   5. High influence
4. What could sport/activity leaders do to ease your concerns with participating in the sport or activity?
   1. Improved education of participants
   2. Improved education of parents
   3. Improved equipment safety features
   4. Better enforcement of rules regarding activities that may lead to concussion
   5. Change of sport rules regarding activities that may lead to concussion
   6. Increased penalties for rule infractions

**SECTION 4 – APPROACH TO EDUCATION**

1. How important to you is awareness and knowledge about sport-related brain injuries/concussions?
   1. Unimportant
   2. Somewhat unimportant
   3. Neutral
   4. Somewhat important
   5. Highly important
2. Who should be responsible for raising awareness and educating the public about sport-related brain injuries/concussions (check all that apply)?
   1. Federal government (e.g. Health Canada)
   2. Provincial government (e.g. health services such as OHIP)
   3. National Sport Organizations (e.g. Hockey Canada or Canada Soccer)
   4. Provincial Sport Organizations (e.g. BC Lacrosse Association or Football Manitoba)
   5. Sports league officials
   6. Sports club officials
   7. Sports team officials
   8. Team coaches or trainers
   9. Education system (Boards of Education and schools)
   10. Your family doctor / GP
   11. The parent of the athlete
   12. Other (please specify)
3. What are the best methods for you to receive information and education about sport-related brain injuries/concussions (check all that apply)?
4. TV ads
5. Radio ads
6. Newspaper ads
7. Newspaper articles
8. Direct mail brochure
9. Brochures distributed by your team, club, league, school, family doctor, community group, etc.
10. Segments about concussion (e.g. how to identify and treat a concussion, profiles of athletes with concussion) during TV news shows
11. Segments about concussionduring TV sports broadcasts
12. YouTube videos
13. Social Media activities (e.g. Facebook, Twitter)
14. Interactive websites
15. Smartphone Apps
16. Other (please specify)

**SECTION 5 – DEMOGRAPHIC INFO**

1. What is your gender?
2. Male
3. Female
4. Prefer not to say
5. What is your age?
   - 1. Under 15
     2. 15-24
     3. 25-34
     4. 35-44
     5. 45-54
     6. 55-64
     7. 65-74’
     8. 75+
     9. Prefer not to say
6. How would you best describe your position (check all that apply)?
7. Athlete
8. Parent or guardian of athlete
9. Community coach
10. Competitive coach
11. High performance coach
12. Sports trainer
13. Team or club personnel
14. Sports league, association or organization personnel
15. Educator, teacher, learning facilitator
16. Medical professional
17. Other (please specify)
18. How would you best describe your ethnicity?
19. Aboriginal Canadian
20. African Canadian/American
21. Asian
22. Caribbean
23. Caucasian
24. Hispanic
25. Middle Eastern
26. South Asian
27. Prefer not to say
28. Other (please specify)
29. What is the highest level of education you have completed?
30. Some high school
31. High school
32. Some post secondary
33. Post secondary (college/university)
34. Masters Degree
35. Doctoral Degree
36. Professional Designation
37. Prefer not to say
38. Other (please specify)
39. What is your annual household income?
40. $0-$20,000
41. $20,001-40,000
42. $40,001-60,000
43. $60,001-80,000
44. $80,001-100,000
45. $100,001-150,000
46. $150,001+
47. Prefer not to say
48. Where do you currently live?
49. Nunavut
50. Northwest Territories
51. Yukon
52. British Columbia
53. Alberta
54. Saskatchewan
55. Manitoba
56. Ontario
57. Quebec
58. New Brunswick
59. Nova Scotia
60. Prince Edward Island
61. Newfoundland& Labrador
62. Outside Canada
63. If you have children under the age of 25, what are their age ranges (check all that apply)?
64. 0-5
65. 6-9
66. 10-12
67. 13-15
68. 16-18
69. 19-24
70. Not applicable

**SECTION 6 – CONTEST ENTRY**

1. Thank you for your time and assistance with this survey. You now have a chance to win a 16GB iPad 2 with Wi-Fi + 3G with a retail value of $649. This contest is open to residents of Canada, excluding the Province of Quebec, who are over the age of majority in the province or territory in which the entrant resides at the time of the contest entry. CLICK TO READ THE FULL CONTEST RULES AND REGULATIONS.
   - 1. Yes I want to enter the contest
     2. No I do not want to enter the contest
2. Please enter your information below. All fields are required for the contest entry.In order to maintain confidentiality, personal info entered in the contest section will NOT be linked or associated with the rest of the respondent's answers.
3. First Name
4. Last Name
5. Email Address
6. Daytime Telephone Number

**FINAL SLIDE**

Thank you! To learn more about sport-related brain injuries and concussion, please visit: <http://www.thinkfirst.ca/programs/concussionqanda.aspx>
